# Three-Dimensional Registration of Freehand-Tracked Ultrasound to CT Images of the Talocrural Joint

**DOI:** 10.3390/s18072375

**Published:** 2018-07-21

**Authors:** Nazlı Tümer, Aimee C. Kok, Frans M. Vos, Geert J. Streekstra, Christian Askeland, Gabrielle J. M. Tuijthof, Amir A. Zadpoor

**Affiliations:** 1 Department of Biomechanical Engineering, Delft University of Technology (TU Delft), Mekelweg 2, 2628 CD Delft, The Netherlands; a.a.zadpoor@tudelft.nl; 2 Orthopaedic Research Center Amsterdam, Academic Medical Centre (AMC), Meibergdreef 9, 1105 AZ Amsterdam, The Netherlands; a.kok@amc.uva.nl (A.C.K.); gabrielle.tuijthof@zuyd.nl (G.J.M.T.); 3 Department of Imaging Science and Technology, Quantitative Imaging Group, Delft University of Technology (TU Delft), Lorentzweg 1, 2628 CJ Delft, The Netherlands; f.m.vos@tudelft.nl; 4 Department of Radiology, Academic Medical Centre (AMC), Meibergdreef 9, 1105 AZ Amsterdam, The Netherlands; g.j.streekstra@amc.uva.nl; 5 SINTEF Medical Technology, 7465 Trondheim, Norway; christian.askeland@gmail.com; 6 Zuyd University of Applied Sciences, Research Centre Smart Devices, Nieuw Eyckholt 300, 6419 DJ Heerlen, The Netherlands; g.j.tuijthof@amc.uva.nl

**Keywords:** talocrural joint, freehand ultrasound, registration, osteochondral defect

## Abstract

A rigid surface–volume registration scheme is presented in this study to register computed tomography (CT) and free-hand tracked ultrasound (US) images of the talocrural joint. Prior to registration, bone surfaces expected to be visible in US are extracted from the CT volume and bone contours in 2D US data are enhanced based on monogenic signal representation of 2D US images. A 3D monogenic signal data is reconstructed from the 2D data using the position of the US probe recorded with an optical tracking system. When registering the surface extracted from the CT scan to the monogenic signal feature volume, six transformation parameters are estimated so as to optimize the sum of monogenic signal features over the transformed surface. The robustness of the registration algorithm was tested on a dataset collected from 12 cadaveric ankles. The proposed method was used in a clinical case study to investigate the potential of US imaging for pre-operative planning of arthroscopic access to talar (osteo)chondral defects (OCDs). The results suggest that registrations with a registration error of 2 mm and less is achievable, and US has the potential to be used in assessment of an OCD’ arthroscopic accessibility, given the fact that 51% of the talar surface could be visualized.

## 1. Introduction

(Osteo)chondral defects (OCDs) of the talocrural joint predispose patients to premature osteoarthritis (OA), if left untreated [1,2,3]. Adequate treatment of OCDs is highly essential, since both OCD and OA adversely affect the patients’ quality of life with joint pain and dysfunction [2]. 

There are various surgical treatment options for talar OCDs including “the excision of the lesion, excision and curettage, excision combined with curettage and drilling/microfracturing, placement of an autogenous bone graft, antegrade drilling, retrograde drilling, osteochondral transplantation and autologous chondrocyte implantation” [4]. The failure rates (i.e., number of unsuccessfully treated patients × 100/number of treated patients) of these treatment strategies can go up to 70% (range 0–70%) [4,5]. The exact reasons for the failure of the treatment are often not completely clear, preventing formulation of effective individual treatment strategies. This could, at least partially, be due to a lack of knowledge on cartilage tissue regeneration in-vivo [6]. To understand the nature of cartilage healing in-vivo and in turn to devise treatment scenarios, longitudinal monitoring of OCDs of patients in short intervals could be helpful [7,8]. 

Ultrasound (US) imaging facilitates frequent evaluation of patients [9] in time, because it is non-invasive, cost-effective, and easily accessible [10]. Recent studies have shown that US could be used to image cartilage and bony abnormalities (e.g., cortex irregularities) [7,11]. Reproducible evaluation of OCDs with US would be, however, challenging. This is because, US images cannot so easily be interpreted and compared over time without having access to the talocrural joint anatomy provided by other modalities, such as computed tomography (CT). To monitor the post-operative tissue regeneration and evaluate any changes in the OCDs as a result of the treatment, US images of the patient could therefore be registered to and used in conjunction with pre-operative CT scans of the same patient. 

In the literature, numerous US to CT bone registration schemes have been previously presented, which include feature- and/or intensity-based techniques [12,13,14,15,16,17,18,19,20,21,22,23,24]. Referring to feature-based techniques, the registration of bone surfaces extracted from both CT and US volumes using a variant of the iterative closest point (ICP) [25] is a common approach. In the study carried out by Muratore et al. [18], ICP technique was used to register vertebral bone surfaces derived from both CT and US volumes. Barratt et al. [12] presented a surface-based registration algorithm that can simultaneously update the freehand tracked US system calibration parameters. Another point-based US to CT registration scheme was described by Moghari et al. [16]. In [16], Unscented Kalman Filter was used to estimate rigid transformation parameters needed for registering two point sets extracted from CT and US images. In the study presented by Brounstein et al. [13], point clouds derived from CT and US volumes were represented as Gaussian Mixture Models (GMM). The registration of two point clouds was realized by minimizing the dissimilarity measure (i.e., L2 distance metric) [13]. In the study [14], the previously proposed GMM surface registration algorithm was improved. Regarding to intensity-based registration techniques, Penney et al. generated probability images from both the CT and US volumes. A normalized cross correlation metric was used as a similarity measure to register the probability images [17]. Winter et al. [19,26] described a surface–volume registration scheme to register US volume with enhanced bone contours to bone surface points culled from CT volume. Nagpal et al. [22] presented a multi-body registration technique, which maximizes the similarity between CT and US data using features derived from the two modalities and voxel intensity information.

All these previously presented registration schemes have been applied to long bones [17,21], vertebrae [19,22,23], and pelvis [16,17,24]. Although, there is no limitation in theory for applying previously described registration techniques to the talocrural joint, none of the studies have focused on this anatomical location. It is therefore not clear how these registration techniques perform individually and relative to each other when dealing with US images of the talocrural joint. Accordingly, a rigid surface–volume registration scheme is presented in this study in order to match CT and freehand-tracked US images of the talocrural joint. The robustness of the registration algorithm to different initialization positions and to the sampling of US data was evaluated on a dataset collected from 12 cadaveric ankles. The presented scheme was also used in clinical practice to evaluate the potential of US imaging as an alternative to a CT scan in pre-operative planning of arthroscopic access to anterior talar OCDs [27]. The contributions of this study are twofold: (1) the talocrural joint is the main focus as an anatomical location, and (2) the question about the potential use of US imaging in clinics has been addressed for the first time, while assessing the performance of the registration scheme. 

## 2. Materials and Methods

**Figure 1:**
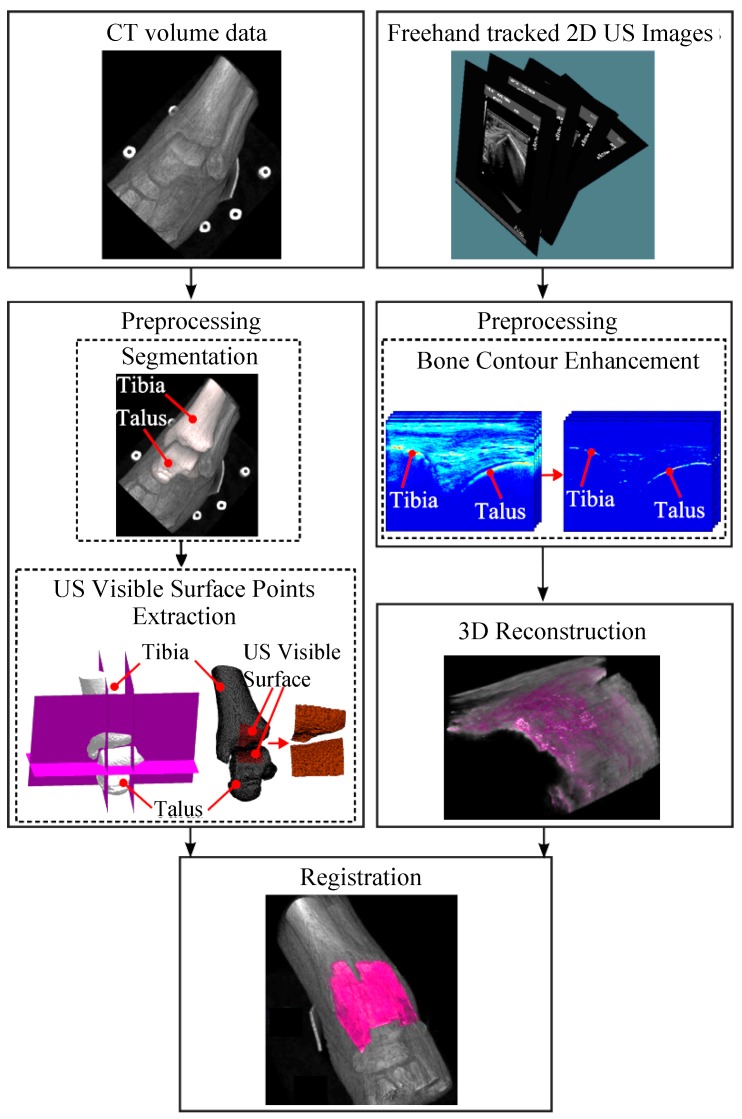
Overview of the rigid surface–volume registration scheme to match computed tomography (CT) and ultrasound (US) images of the talocrural joints. CT and US images are pre-processed before the registration. During the pre-processing step, surfaces of bones (i.e., tibia and talus) that can be visualized with US in maximal-plantar flexed ankle are extracted from the CT image and bone contours in freehand tracked 2D US images are enhanced using intensity invariant local-phase based approach and bone shadow information. The 3D bone response data is reconstructed from 2D enhanced US images using the position of the US probe that had been recorded with an optical tracking system. Registration is initialized at a location defined using the position of the six fiducials in the US and CT spaces.

An overview of the registration scheme is presented in Figure 1. Initially, freehand-tracked 2D US images and a CT scan of the talocrural joint of the same patient are acquired. In the pre-processing step of the CT scan, a point set is extracted from the distal tibial and talar bone surfaces. Simultaneously, bone contours in 2D US data are enhanced based on the monogenic signal representation of 2D US images [28]. Subsequently, 3D US image with enhanced bone contours is reconstructed from the preprocessed 2D US data and using the position of the conventional US probe that was recorded with an optical tracking system. During the registration of the surface extracted from the CT scan to the monogenic signal feature volume, six transformation parameters (three for rotation and three for translation) are estimated so as to optimize the sum of monogenic signal features over the transformed surface extracted from the CT data. The CT and US data are considered as “fixed” and “moving” images, respectively, during the registration.

In the following paragraphs, the methods used for acquisition of freehand-tracked 2D US images and CT scan of the talocrural joint are provided first. Subsequently, the steps in the proposed registration scheme are extensively described. Then, the details of the tests performed on the acquired data to evaluate the precision of the registrations are provided. Finally, the methodology applied in a clinical study is presented. 

### 2.1. Test Data Acquisition

A test dataset consisting of CT scans and freehand-tracked 2D US images of 12 cadaveric ankles was collected at Academic Medical Center (AMC, Amsterdam, The Netherlands). For the use of the cadaveric ankles, permission from the Medical Ethical Committee of AMC was not needed. 

**Figure 2:**
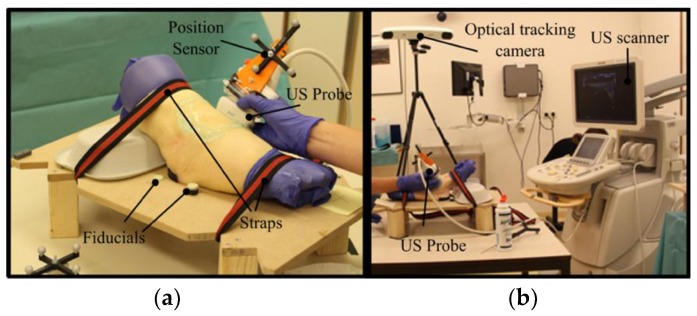
Experimental settings in the ultrasound room. (**a**) Prior to CT imaging and US sweeping, each cadaveric ankle was placed on a foot plate in maximal plantar flexion and was tightened using straps. The US probe on which the position sensor mounted was slowly swept over the cadaveric ankles and the position of the probe was recorded using (**b**) the optical tracking camera.

Each cadaveric ankle was tightly fixed on a foot-plate in maximal plantar flexion with straps to avoid any movement between acquisition of CT scan and US sweeps (Figure 2a). Six fiducial markers were attached to the foot-plate (i.e., four markers on top of the foot-plate and two markers underneath), which are visible in the CT scan. These markers were pointed out with an external pointer and recorded during the US data acquisition (Figure 2a).

#### 2.1.1. CT Scans

CT scans were acquired using a Philips Brilliance 64 CT scanner (Philips Healthcare, Best, The Netherlands). The acquisition parameters were: effective dose 150: mAs/slice, rotation time: 0.75 s per 360°, pitch: 0.875, slice thickness: 0.6 mm, and ultra-high-resolution mode. Tomographic reconstructions were made with a field of view of 154 mm, a slice increment of 0.3 mm, and a matrix of 512 × 512 pixels. The voxel sizes were 0.3 mm × 0.3 mm × 0.3 mm.

#### 2.1.2. Freehand Tracked 2D US Images

For the US data acquisition, an iU22 xMatrix scanner (Philips Healthcare, Best, The Netherlands) (Figure 2b) was used together with a 17- to 5-MHz broadband linear array probe (Philips Healthcare, Best, The Netherlands) (Figure 2a). The position of the US probe was recorded by the means of a Polaris optical tracking system (Northern Digital Inc., Waterloo, Ontario, Canada) (Figure 2b), by mounting a passive reflective marker to the US probe that was followed by the tracking camera (Figure 2a) (3D root mean square volumetric accuracy of the position sensor ≤ 0.25 mm). 

The navigation system CustusX (SINTEF, Trondheim, Norway) [29], which allows for connections between the US scanner and the optical tracking system, was employed for data acquisition and reconstruction of the 3D US data from 2D US images. Prior to US data acquisition, the US probe was calibrated (calibration error = 0.23 mm) using a point target phantom [30].

The US imaging parameters (e.g., depth, gain) were set by an experienced radiologist specialized in musculoskeletal ultrasound imaging. For each cadaveric ankle, two different sweep types, namely “Sweep Type I” and “Sweep Type II”, were performed. In the “Sweep Type I”, the US acquisition started at the medial side of the talocrural joint with the probe placed perpendicular to the foot, on the deltoid ligament. The US probe was moved from the medial side to the lateral rim of the talus in such a way that the tibial rim and the dorsal surface of the talus were constantly visualized. The “Sweep Type II” involved positioning the probe slightly more proximal, supplemented with a return sweep back to the starting position on the medial side with the probe positioned more distally at the lateral rim of the talus. During each acquisition, the US probe was moved slowly (i.e., minimum of twenty seconds spent) over a cadaveric ankle while aiming to keep complete contact between the US probe and the cadaveric skin. After both sweeps, the position of the six fiducials was recorded using a calibrated pointer and the CustusX software.

### 2.2. Data Preprocessing

#### 2.2.1. Surface Point Extraction from CT Data

Both talus and distal tibia were segmented from each CT scan using Mimics (version 14.01, Materialise, Leuven, Belgium) to yield triangulated surfaces. During the segmentation of the bones, a similar procedure as described by Tümer [31] was followed.

Regions on the triangulated bone surfaces that can be visualized with US (Figure 1) were defined and isolated from the rest using custom-made code developed in Matlab (Matlab 2013b, The Mathworks Inc., Natick, MA, USA) (details in Supplementary Material). The vertices of the triangles located on the isolated surfaces were extracted.

#### 2.2.2. Bone Surface Enhancement in Ultrasound

**Figure 3:**
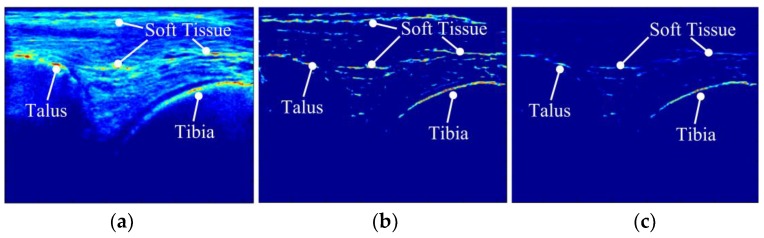
Bone contour enhancement in a 2D US image. (**a**) An original 2D US image (**b**) Phase Symmetry (*PS*) map calculated based on monogenic signal representation of the 2D US image. (**c**) Bone response map obtained based on the product of *PS* and the shadow values (*SH*).

Bone contours in 2D US images were enhanced using an intensity-invariant local-phase technique [32,33,34,35]. Following [34], a local phase-based feature detector “Phase Symmetry (PS (x,y))”, which is sensitive to the ridgelike edges observable at the bone boundaries in the US images was used. The *PS* was defined based on the monogenic signal representation [36] of 2D US images (Figure 3a) (details in Supplementary Material). As *PS* has high response at symmetry locations [37], other features such as soft tissue-muscle interface were also enhanced together with bone contours (Figure 3b). To highlight the bony contours further, bone shadow information [37] was taken into account. Briefly, the weighted sum of intensity values of pixels extending from a pixel of interest was used to quantify the shadow:

(1) $$SH\left(x,y\right)=\;\frac{\sum_{j=x}^{H}G\left(j,y\right)U\left(j,y\right)}{\sum_{j=x}^{H}G\left(j,y\right)},$$

where SH(x,y) is the shadow value for a pixel at row x and column y of the US image. H and G(.) represent the total number of rows of the image U(x,y) and the Gaussian weighting function, respectively. Combining PS and shadow information, the bone responses BR(x,y) [37] (Figure 3c) that represent 2D US image with enhanced bone contours were obtained:

(2) BR(x,y)=PS(x,y)·SH(x,y),

### 2.3. 3D Bone Response Data and US to CT Registration 

A 3D bone response data was reconstructed from 2D enhanced US images (i.e., BR) using the position information of the US probe. 

For each of 24 US sweeps (i.e., 12 cadaveric ankles × 2 sweep types), the registration was initialized at a location defined using the position of the six fiducials in the US and CT spaces. 

The objective function, which was optimized during the registration, was the sum of the bone response values over the transformed surface extracted from the CT data:

(3) $$f\left(\alpha ,\;\beta ,\;\gamma ,\;\Delta_{x},\Delta_{y},\Delta_{z}\right)=f\left(x\right)=\;\overset{N}{\underset{i=1}{\sum }}BR\left(R_{\alpha \beta \gamma }\cdot p_{i}+{\left(\Delta_{x},\Delta_{y},\Delta_{z}\right)}^{T}\right),$$

where N is the total number of sampling points p lying on the surface extracted from the CT data, $R_{\alpha \beta \gamma }$ is the rotation matrix that depends on the angles α, β, and γ (i.e., rotation parameters expressed in rad), and $\Delta_{x}$, $\Delta_{y}$ and $\Delta_{z}$ are the translational parameters in mm.

Six transformation parameters (i.e., three for rotation and three for translation) were estimated using the Covariance Matrix Adaptation Evolution Strategy (CMA-ES) [38], as the technique can be used for medical image registration tasks [26] and evolutionary algorithms have been reported to be robust [26,39,40]. During the optimization, a box constraint was applied in a way that the transformation parameters stayed in the range of ±0.2 rad (±11.5°) and ±5 mm surrounding the initialization location (i.e., α, β, γ, $\Delta_{x}$, $\Delta_{y}$ and $\Delta_{z}$ were all equal to zero at the start of the registration).

A similar stopping criterion was applied as described by Winter et al. [26]. Registrations were stopped, when the relative progress of the best individuals over the last 10 generations dropped below the threshold, $f_{thr}=\;10^{-6}$. With the aim of decreasing the possibility of registration algorithm to get stuck in local maxima, multistart optimization was performed. The optimization algorithm was restarted once the stopping criterion was met from the same initial position together with all the strategy parameters of the CMA-ES reset to their initial values [26].

### 2.4. Evaluation of the Registrations

A reference registration for each cadaveric ankle was identified in a similar way as described by Winter et al. [26]. For each of the 24 US sweeps, the registration algorithm was run 20 times, following an initialization at a location defined using the position of the six fiducials in the US and CT spaces. Among the 20 registrations per sweep, the one having the highest fitness value was picked as the reference registration. The validity of the reference registrations was visually checked.

The registration algorithm was run 100 times for each US sweep with initial misalignments (i.e., Test I). Taking each of the 24 US sweeps into account, a set of 100 initialization positions was determined by transforming the reference registration to a position away from its assumed optimum. The transformations were randomly created using a uniform distribution with rotation and translation parameters ranging between $\pm \;0.2\;\mathrm{rad}\;\left(\pm 11.5^{\circ }\right)$ and ± 5 mm, respectively. The initial position and the position of a volume after registration were compared to the position in the defined optimum (i.e., $di_{RMS}$, $df_{RMS}$) by calculating the root-mean-square (RMS) target registration error (TRE) [17]:

(4) $$df_{RMS}=\sqrt{\frac{1}{N}\sum_{x}∥T_{\mathrm{REG}}x-T_{REF}x{∥}^{2}},$$

where **x** is a point set extracted from 3D US data and *N* is the number of points in the set. $T_{REG}$ and $T_{REF}$ represent transformation matrices obtain as a result of a registration and as a result of the reference registration.

To assess the effects of the 3D US volume “density” on the performance of the registration algorithm (i.e., Test II), the amount of collected 2D US slices in each US sweep was decreased 25% by randomly leaving out slices. The registration algorithm was run 100 times for each US sweep starting from the same initial position using 75% of the full data. The position of a volume after registration were compared to the position in the defined optimum ($df_{RMS}$) according to Equation (4).

During evaluation of the Test I and II results, registrations having $df_{RMS}$ values higher than 2 mm were considered as failures.

### 2.5. Clinical Study

**Figure 4:**
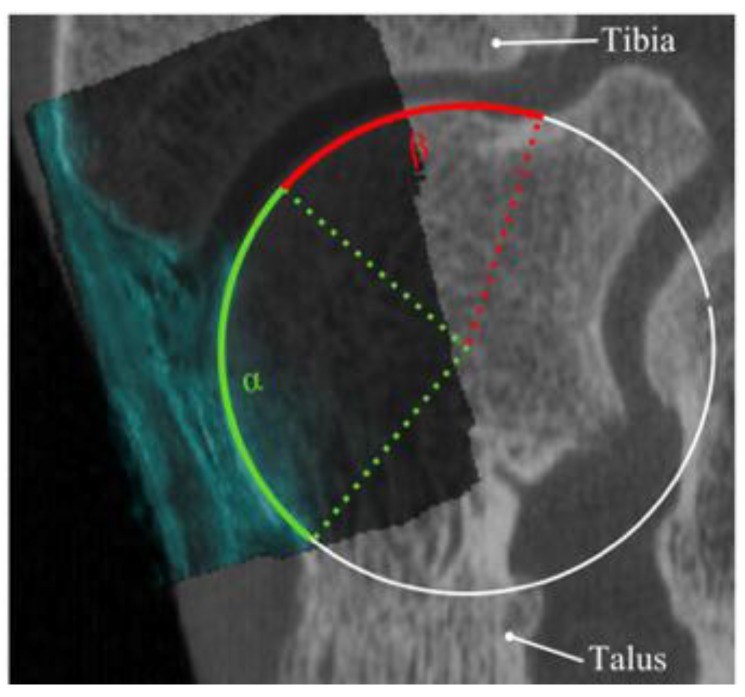
Sagittal view of one of the US volumes registered to the CT scan. The visible cartilage and cartilage covered by the tibia are represented, respectively, with *α* and *β.* The percentage of the visible cartilage was defined as the ratio of *α* to *α + β* (i.e., the total cartilage surface).

To understand whether US imaging could be an alternative to a CT scan in pre-operative planning of arthroscopic access to anterior talar OCDs, the percentage of the talar cartilage surface that could be visualized with US imaging was determined following a similar approach as described by van Bergen [41]. Two observers analyzed the US data of 12 cadaveric ankles that were registered to the CT scans of the same cadaveric ankles using the proposed registration scheme. Each observer, using a custom-made code [41] developed in Matlab (Matlab 2016b, The Mathworks Inc., Natick, MA, USA), defined the percentage of the visible talar dome contour (i.e., the arc length of the anterior talar dome, α divided by the arc length of the complete talar dome, α+β) (Figure 4) on 2D slices extracted from the lateral, central, and medial aspects of the talus. To evaluate the intraobserver reliability, the data was assessed one more time by one of the observers. Average percentages of the visible talar dome contour were calculated for each location and for each observer.

## 3. Results

**Figure 5:**
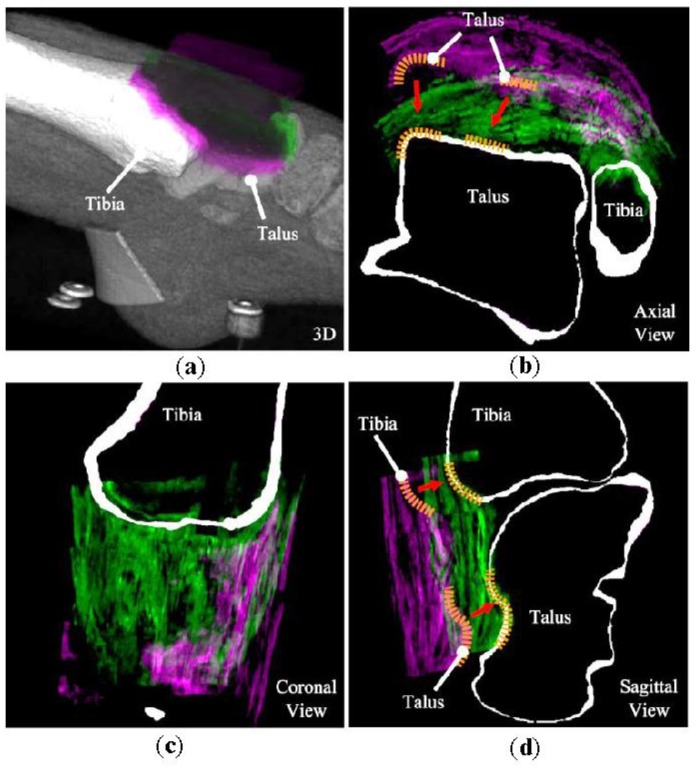
A visualization of one of the successful registrations achieved in Test I. (**a**) the 3D view (**b**) Axial view (**c**) Coronal view and (**d**) Sagittal view. US volume in purple color represents the data at initialization position prior to registration. US volume in green depicts the result of the registration. Dashed lines in yellow highlight the contours of the tibia and talus observed in both axial and sagittal views. Arrows in red shows that US volume goes from its initial position (i.e., US volume in purple) to its final position (i.e., US volume in green).

An example from Test I, which depicts the initial misalignment and the final registration is given in Figure 5. The visualization shows that it is possible to register US to CT images of the talocrural joint quite well using the proposed registration scheme.

Table 1 shows the number of successful registrations achieved in Test I and the mean of the $di_{RMS}$ values together with $df_{RMS}$ averaged over all successful registrations. The success rate across all the US sweeps in ‘Sweep Type I’ and ‘Sweep Type II’ were, respectively, 46% and 59%. The mean of the $df_{RMS}$ values averaged over all successful registrations in “Sweep Type I” and “Sweep Type II” were, respectively, 0.9 mm ± 0.5 mm and 0.8 mm ± 0.3 mm.

**Figure 6:**
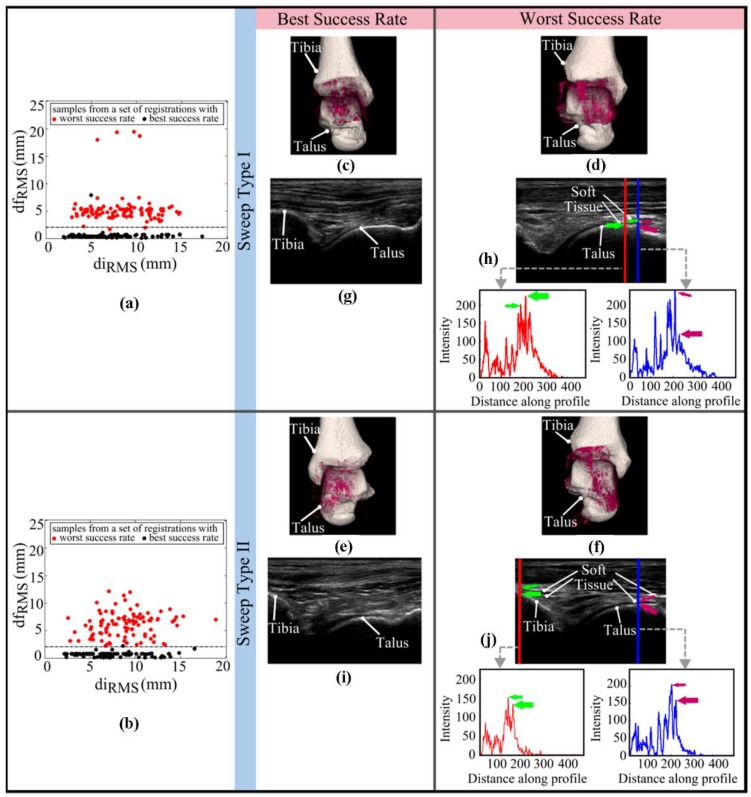
Plots of the $df_{RMS}$ values vs. the $di_{RMS}$ values calculated over 100 registrations are given for (**a**) the ‘Sweep Type I’ US sweeps (**b**) the ‘Sweep Type II’ US sweeps showing the best and worst success rate in Test I. Black (*n* = 100) and red (*n* = 100) dots in (**a**) are samples retrieved from a set of registrations performed for the ‘Sweep Type I’ US sweeps with the best and worst success rate, respectively. In a similar manner, those ($n_{black}$ *=* 100 and $n_{red}$ = 100) in (**b**) are related to a set of registrations run for the ‘Sweep Type II’ US sweeps with the best and worst success rate. Visualizations of 3D bone response volumes of the ‘Sweep Type I’ US sweeps (**c**) with the best and (**d**) the worst success rate, and of the ‘Sweep Type II’ US sweeps (**e**) with the best and (**f**) the worst success rate in the Test I are shown. Original 2D slices taken from the US sweeps (**c**–**f**) are presented in a corresponding manner (**g**–**j**). The intensity profiles of the two vertical lines (i.e., intensity vs. distance along profile graphs shown in red and blue correspond to the lines drawn in red and blue, respectively) displayed in (**h**) and (**j**) indicate that the ridge-like edge feature corresponding to the expected bone boundary can be weaker than those related to the soft tissue interface. Thin and thick arrows shown in green and purple point to the ridge edge features linked, respectively, to soft tissue interface and bone boundary.

Results of the Test I are displayed as plots of $df_{RMS}$ vs. $di_{RMS}$ for the US sweeps showing the best (i.e., 99% for both the “Sweep Type I” and “Sweep Type II”) and worst success rate (i.e., 1% for the “Sweep Type I” and 0% for the “Sweep Type II”) at a total of 100 registrations each (Figure 6a,b). No clear relationship could be detected between the registration failure and the distance of the initial point from the defined reference. Registered bone surfaces and 3D bone response volumes given for the US sweeps with the best and worst success rate in Test I (Figure 6c–f) show that similar regions in each sweep type were scanned during the US data acquisition. Regarding the raw 2D US slices extracted from the “Sweep Type I” and “Sweep Type II” sweeps with the worst success rate (Figure 6h,j), soft tissues seem to stand relatively closer to the bony contours as compared to those seen in the ones obtained from the US sweeps with the best success rates (Figure 6g,i). The scanline profiles presented in Figure 6h,j show the intensity values of the pixels crossed by the two vertical lines (red and blue). Based on these profiles, it could be noted that soft tissues surrounding the bone may have higher intensity values as compared to those of the bony contours.

Table 2 shows the number of successful registrations achieved in Test II and the mean of the $df_{RMS}$ values averaged over all successful registrations for each of 24 US sweeps. The average success rate was 47% and 58%, respectively, in “Sweep Type I” and “Sweep Type II”. The mean of the $df_{RMS}$ values averaged over all successful registrations in “Sweep Type I” and “Sweep Type II” were, respectively, 0.7 mm ± 0.4 mm and 1.0 mm ± 0.6 mm. For 1 out 12 US sweeps with reduced “density” in “Sweep Type II” registrations consistently failed, while it was the case for 5 US sweeps in “Sweep Type I” (Table 2).

The results of the clinical case in terms of the mean percentage of visible talar surface at the lateral, central, and medial part of the talus, are presented in Table 3. On average, 51 ± 3% of the talar surface of each maximum plantar flexed cadaveric ankle could be imaged using US (Table 3).

## 4. Discussion

The goal of this study was to register freehand-tracked 2D US images of the talocrural joint to CT images. To this end, a rigid-surface volume registration scheme was presented and the performance of the registration algorithm was tested on a dataset collected from 12 cadaveric ankles. The registration algorithm was also used for a practical problem to evaluate whether US imaging can be a substitute of CT in assessment of accessibility to an OCD by anterior arthroscopy.

Visual inspection of the reference registrations and the example use of the registration algorithm confirm that multi-modality image registration is possible for a talocrural joint and satisfactory registration results (Figure 5) could be achieved with the proposed scheme.

Concerning Test I, the registration algorithm had varying levels of success (Table 1). The large variance observed in the registration rate cannot directly be attributed to various initialization positions with a certain distance away from the defined reference. Differences in the performance of the registration algorithm to compensate similar mean $di_{RMS}$ values (e.g., the success rate of 99% and 1% achieved in “Sweep Type I” of two different cadaveric ankles with the mean $di_{RMS}$ values of 7 mm) (Table 1) suggest that the success rate of the algorithm is dependent on the acquired US data. Comparing raw 2D US images of the US sweeps showing the best (i.e., 99% for both the “Sweep Type I” and “Sweep Type II”) and worst success rate (i.e., 1% for the “Sweep Type I” and 0% for the “Sweep Type II”) in the Test I (Figure 6g–j), it was observed that soft tissues are positioned relatively closer to the bone contours and show more bony-like features in the most failed US sweeps (Figure 6h,j). With a close look at the scanline profiles of both vertical lines (red and blue) shown in Figure 6h,j, it becomes clearer that dominant ridge edge responses do not always correspond to the expected bony location, but may represent the high intensity soft tissue interfaces. In the current study, commonly-used local phase based descriptor optimized for ridge detection (i.e., *PS*) [13,34] has been used to extract bony contours present in US images. As the *PS* detector does not provide or correct the responses at locations where the assumed feature model, i.e., ridge-like edge for bone, is violated and the registration algorithm is not capable of distinguishing between soft and bony tissues, it is possible that the registrations could not always end in correct positions due to the existence of bony-like information in some of the 3D volumes (Figure 6d,f). In the future, more sophisticated descriptors, such as the local phase tensor [28], are suggested to be used to determine whether an improvement in bone detection and in the performance of the registration algorithm could be achieved in the US sweeps consisting of different feature types.

The results of the Test II indicate that the quality of the acquired US data plays an important role in the success of the registration process. Decreasing the number of slices in US sweeps seems to adversely affect the performance of the registration algorithm (Table 2). Reduction in the amount of information on bones in 3D bone response volumes could well explain the relatively lower performance of the registration algorithm. Moreover, in both tests, the mean success rate was higher in “Sweep Type II” as compared to those in “Sweep Type I”. This finding can be explained by relatively larger scanned area of the talar bone in “Sweep Type II”. Increase in the amount of data on bony contours could help the registration algorithm to more easily find the optimum position.

Both “Sweep Type I” and “Sweep Type II” seem to be adequate to image approximately 50% of the anterior talar dome (Table 3). This is comparable to the area defined previously as the part of the talar dome accessible with anterior arthroscopy [41]. The results of the clinical test therefore suggest that US could be used to assess whether an OCD can be accessed by anterior arthroscopy.

Although, the freehand-tracked 2D US images of 12 cadaveric ankles were acquired by the experienced operator and using a protocol that had been included in previous studies [7,8], the results of the tests and the observations made on US sweeps suggest that there is a room for improvements in the US data acquisition phase. One of the changes that could be made is the use of an alternative good quality US probe specially designed/fine-tuned for musculoskeletal applications. A US probe with a relatively smaller head size (e.g., L15-7io, Philips Healthcare, Best, The Netherlands) may allow the operator to more easily orient the probe to maintain it at a perpendicular position with respect to the bones. With an enhancement in the positioning of the US probe on the ankle joint, the chance of getting sharp bone boundary interfaces and decreasing the amount of unwanted information on soft tissues around the bones may be increased. A closer study of the 3D bone response volumes of the US sweeps that displayed the best and worst success rate in the Test I (Figure 6c–f) showed that the variances observed in the success rates of the registration algorithm do not originate from any specific region on talus or distal tibia. Moreover, the success rate is expected to increase, if the US sweeps contain more information regarding the edges of the target bones. The scanned area is recommended to be increased in the future studies and includes the regions beyond the visible cartilage surfaces, e.g., by starting and finishing the data acquisition until the malleoli are visualized.

In addition to enhancing the US data acquisition protocol, the presented registration scheme could be further improved by providing real-time feedback to the operator, thereby guaranteeing successful registration in the clinical settings. In the current study, the computation time for the pre-processing of a single 2D US slice and the registration of a 3D US bone response volume to a surface extracted from a CT scan is ≈4 s and ≈3 min, respectively. To provide real-time guidance, the computational time has to be decreased, for example, through optimizing the code and/or implementing the methods on a multi-processor graphic processing unit (GPU).

To determine “gold standard” registrations and quantitatively evaluate the accuracy of the registrations, data could be collected from bones into which fiducials have been implemented. Fiducials were not attached to the bones to keep the conditions as close to the clinical reality as possible. To delineate a “gold standard”, another solution could be the use of anatomical landmarks. Although we made an attempt to define points on the talus that could be consistently found in all individuals and be used as anatomical landmarks, no such points could be ultimately found. Neither could we find any studies in the literature that could be referred to for the use of anatomical landmarks on the talus. It is therefore not clear to what extent the idea of using anatomical landmarks is practically feasible.

## 5. Conclusions

To the best of our knowledge, this is the first study proposing a scheme to register freehand-tracked 2D US images of a talocrural joint to the CT scan of the same joint. Results of the study showed that multi-modality image registration is possible for the talocrural joint and satisfactory registration results could be achieved with the proposed method. This could enable anatomical correlation of US images based on CT scan, thereby improving the possibilities for retrospective analysis and prospective follow-up of patients with diseases at the talocrural joint. The retrospective and prospective clinical studies could help gain knowledge in cartilage healing in-vivo and in turn formulate effective patient-specific treatment strategies. Further research, however, needs to be performed before clinical implementation of the proposed method is possible. The presented registration scheme could be enhanced in particular by using a more sophisticated approach to the pre-processing of US images, modifying the protocol established for the US data acquisition and implementing the methods in such a way that real-time feedback could be provided to guide the operator.

## Figures and Tables

**Table 1:** Number of successful registrations and the mean root-mean-square (RMS) TRE (i.e., mean $di_{RMS}$ and mean $df_{RMS}$ ) values for each US sweep averaged over all successful registrations in Test I. For each registration, $di_{RMS}$ and $df_{RMS}$ describe how far a volume is away initially and after registration from its assumed optimum, respectively.

US Sweep	Number of Successful	Mean (and STD) (mm)	
Type	Registrations	*d_iRMS_*	*d_fRMS_*
I	99	6.96 (3.27)	0.46 (0.17)
	80	7.50 (2.87)	1.24 (0.62)
	71	6.99 (2.49)	1.80 (0.18)
	65	8.72 (3.78)	0.62 (0.28)
	46	9.66 (4.66)	0.51 (0.17)
	39	7.43 (2.46)	0.57 (0.35)
	37	10.60 (3.79)	0.70 (0.54)
	35	6.97 (2.12)	0.76 (0.68)
	29	6.95 (2.73)	0.57 (0.36)
	24	10.04 (3.64)	0.65 (0.67)
	20	8.09 (2.66)	0.81 (0.62)
	1	7.04 (0.00)	1.69 (0.00)
II	99	7.28 (3.06)	0.66 (0.32)
	97	8.04 (3.45)	0.93 (0.30)
	91	8.55 (3.30)	0.26 (0.41)
	89	8.33 (3.42)	1.21 (0.57)
	78	8.55 (3.88)	0.95 (0.36)
	77	8.67 (3.66)	0.43 (0.44)
	76	7.35 (3.01)	1.33 (0.14)
	35	9.50 (2.95)	0.82 (0.43)
	28	7.97 (2.84)	0.62 (0.45)
	24	9.73 (3.96)	0.64 (0.43)
	16	9.41 (4.03)	0.68 (0.35)
	0	-	-

**Table 2:** Number of successful registrations and the mean RMS TRE (i.e., mean $df_{RMS}$ ) values for each US sweep averaged over all successful registrations in Test II. For each registration, $df_{RMS}$ describes how far a volume is away after registration from its assumed optimum.

US Sweep	Number of Successful	Mean (and STD) (mm)
Type	Registrations	*d_fRMS_*
I	100	0.31 (0.19)
	100	1.38 (0.28)
	99	0.62 (0.33)
	98	0.63 (0.26)
	90	0.94 (0.47)
	77	0.75 (0.29)
	2	0.40 (0.33)
	0	-
	0	-
	0	-
	0	-
	0	-
II	100	0.35 (0.27)
	100	0.47 (0.29)
	100	0.56 (0.20)
	100	0.60 (0.25)
	99	0.61 (0.32)
	62	0.47 (0.36)
	52	1.63 (0.18)
	31	1.42 (0.41)
	26	1.05 (0.32)
	22	1.91 (0.08)
	3	1.88 (0.06)
	0	-

**Table 3:** The average percentage of the talar surface that could be visualized with US from two observers for the lateral, central and medial part of the talus. Observer 1 served as an internal control by performing the analysis twice: Observer 1(1) and Observer 1(2).

	Visible Talar Surface (% of the Total Talar Surface)		
	Lateral	Central	Medial
**Observer 1(1)**	55.3 (47.2–69.1)	47.9 (42.3–58.2)	51.7 (42.0–62.7)
**Observer 1(2)**	55.4 (46.3–68.1)	47.3 (38.2–59.0)	52.4 (42.2–61.6)
**Observer 2**	53.6 (42.8–66.1)	47.3 (36.8–60.0)	51.4 (42.5–59.0)

## Supplementary Materials

The following are available online at http://www.mdpi.com/1424-8220/18/7/2375/s1.
