# Supplementary material for: Three-Dimensional Registration of Freehand-Tracked Ultrasound to CT Images of the Talocrural Joint
Source: Sensors (Basel). 2018 Jul 21;18(7):2375. doi: 10.3390/s18072375 (PMC6068753; doi:10.3390/s18072375)
Supplement: Supplementary file 1 [file sensors-18-02375-s001.pdf]

SUPPLEMENTARY MATERIAL

Monogenic Signal, Local Phase and Phase Symmetry Measure:

To perform local analysis, a complex analytic signal $f_A(x)$ can be used and be formed by taken the original signal $f(x)$ (i.e. the real part) and its Hilbert transform $H[f(x)]$ (i.e. the imaginary part) into consideration:

$$f_A(x) = f(x) + iH[f(x)], \quad (1)$$

The local phase $\phi(x)$ can be computed from the analytic signal:

$$\varphi(x) = \tan^{-1}\left(\frac{H[f(x)]}{f(x)}\right), \quad (2)$$

Hilbert transform is mathematically restricted to a 1-D function. Felsberg and Sommer presented the monogenic signal [1], which is an extension of the analytic signal from 1-D to *N*-D by means of the Riesz transform.
The monogenic signal $f_M(x,y)$ in 2-D can be written in the same format as the 1-D analytic signal:

$$f_M(x,y) = f_e(x,y) + if_R(x,y) \quad (3)$$

where, f_e is the even component of the original 2-D image calculated by convolving the original image with an isotropic band-pass filter (e.g. log-Gabor filter), f_R represents the odd components of the band-pass filtered image:

$$f_e(x,y) = even(x,y) = f(x,y) * g(x,y) \quad (4)$$

$$f_R(x,y) = odd(x,y) = \sqrt{(f(x,y) * g(x,y) * h_1(x,y))^2 + (f(x,y) * g(x,y) * h_2(x,y))^2}$$

where, g(x,y) is the spatial domain representation of an isotropic band-pass filter $G(u,v)$; $h_1(x,y)$ and $h_2(x,y)$ are the spatial domain representation of the Riesz filter' fourier domain representation $H_1(u,v)$ and $H_2(u,v)$:

$$\left(H_1(u,v), H_2(u,v)\right) = \left(i\frac{u}{\sqrt{u^2+v^2}}, i\frac{v}{\sqrt{u^2+v^2}}\right) \quad (5)$$

Referring to the study of Hacihaliloglu et al. [2], a ridge-like edge appears at bone boundaries in US images. Therefore, in this study, a monogenic signal based multiscale ridge detector $PS(x,y)$(i.e. Phase Symmetry) that is sensitive to bone surface localization in US images is calculated in a similar way described by Hacihaliloglu et al. [2,3]:

$$PS(x,y) = \sum_{sc} \frac{\lfloor [|even_{sc}(x,y)| - |odd_{sc}(x,y)|] - T_{sc} \rfloor}{\sqrt{even_{sc}{}^2(x,y) + odd_{sc}{}^2(x,y) + \varepsilon}} \quad \textbf{(6)}$$

where, *sc* stands for the scale variable and ε is a small constant to avoid division by zero. The $\lfloor . \rfloor$ operator indicates that any negative values are replaced by zero. T_{sc} is a scale-specific noise threshold value [4].

Surface Point Extraction from CT Data

A point set from each pair of adjacent bones (i.e. distal tibia and talus) surfaces, which can partly be visualized using US, were obtained as follows:

1. Dicom images of a cadaveric ankle were imported in Matlab (Matlab 2013b, The Mathworks Inc., Natick, MA) and users were let scrolling down through slices (i.e. slices in sagittal view) to pick a slice in which the talar head – neck transition region (Figure 1) can clearly be seen.

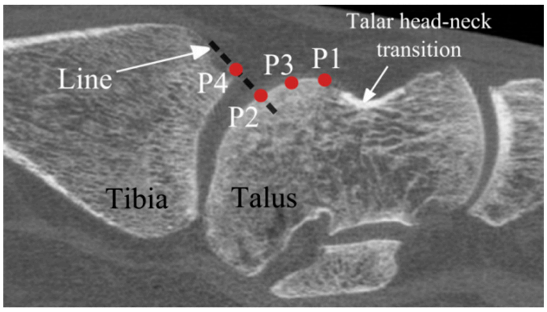

Figure 1. Sagittal view of the ankle joint. Three points 'P1-P3' on talus and one point 'P4' on distal tibia are determined by users. Black dash line represents a fictive line passing through the utmost subchondral part of the tibia and it is used in determination of points 'P2' and 'P4'.

2. Users were let to pick four different points (i.e. three points on talus and one point on distal tibia) (Figure 1). The first point on talus is located close to the talar head-neck transition region. The second point on talus is positioned at the intersection point of a fictive line passing through the utmost subchondral part of the tibia with the talar dome (Figure 1). The third point on talar dome is defined in a way that it lies in between two previously created points (Figure 1). The fourth point is marked on the utmost subchondral part of the distal tibia (Figure 1).
3. Users were second time let to pick a slice by scrolling down through slices (i.e. slices in axial view). A slice is determined in a way that the talar dome width approximately gets its maximum (Figure 2).
4. Users were enabled to pick two different points at the outermost points of the talar dome (Figure 2).

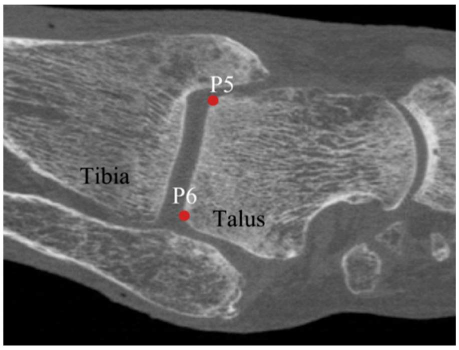

Figure 2. Axial view of the ankle joint. Two Points 'P5' and 'P6' are determined at the outermost locations of the talar dome in the slice, in which the talar dome width roughly gets its maximum.

5. Normals of the planes that pass through the points 'P1', 'P3', 'P5' and 'P6' are determined as follows:
   5.1. **Plane 1** (i.e. it passes through the point 'P1') (Figure 3): A circle is created using the points 'P1', 'P2' and 'P3'. Generated circle is discretized with 1000 points. One of the points, which is the nearest by the point 'P1' is chosen and named 'P7'. The normal of the 'Plane 1' points from the point 'P1' to the point 'P7'.
   5.2. **Plane 2** (i.e. it passes through the point 'P3') (Figure 3): A line is drawn using the points 'P3' and 'P4' and it is rotated by 90°.
   5.3. **Plane 3** (i.e. it passes through the point 'P5') (Figure 3) and **Plane 4** (i.e. it passes through the point 'P6') (Figure 3): Normals are defined in a way that they point outward from the respective face of talus.
6. Planes are drawn using the points 'P1', 'P3', 'P5' and 'P6' and the normals defined in the previous step (Figure 3). Triangulated bone (i.e. distal tibia and talus) surfaces, which have been extracted from the segmentation results, are cut using the four planes 'Plane 1 - Plane 4' and surfaces remaining between four planes are isolated (Figure 3).
7. Vertices of triangles located on the isolated surfaces (Figure 3) are extracted and used as sampling points during registration.

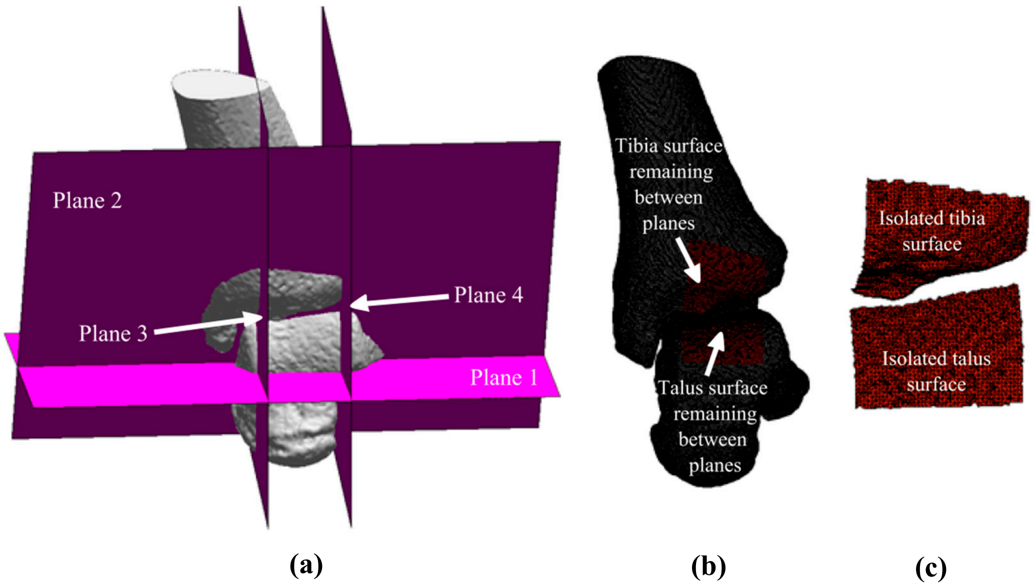

Figure 3. (a) Four planes passing through the points 'P1', 'P3', 'P5' and 'P6' are generated. **(b)** Bone surfaces remaining between four planes are determined. **(c)** Surfaces remaining between four planes are isolated and vertices of the triangles located on isolated surfaces are used as sampling points in registration.

REFERENCES

[1] Felsberg, M.; Sommer, G.; Felsberg, M.; Sommer, G. The Monogenic Signal. *IEEE Trans. Image Process.*, **2001**, *49*, 3136–3144.

[2] Hacihaliloglu, I.; Abugharbieh, R.; Hodgson, A.J.; Rohling, R.N. Bone Surface Localization in Ultrasound Using Image Phase-Based Features. *Ultrasound Med. Biol.*, **2009**, *35*, 1475–1487.

[3] Hacihaliloglu, I.; Rasoulian, A.; Rohling, R.N.; Abolmaesumi, P. Local Phase Tensor Features for 3-D Ultrasound to Statistical Shape+pose Spine Model Registration. *IEEE Trans. Med. Imaging*, **2014**, *33*, 2167–2179.

[4] Kovesi, P. Image Features from Phase Congruency. *Videre*, **1999**, *1*, C3–C3.
